# Comprehensive elucidation of the differential physiological kale response to cytokinins under in vitro conditions

**DOI:** 10.1186/s12870-024-05396-8

**Published:** 2024-07-15

**Authors:** Monika Kamińska, Agata Styczynska, Anna Szakiel, Cezary Pączkowski, Agata Kućko

**Affiliations:** 1 Department of Plant Biochemistry, Faculty of Biology, University of Warsaw, Miecznikowa 1, Warsaw, 02-096 Poland; 2 Department of Plant Physiology, Institute of Biology, Warsaw University of Life Sciences- SGGW (WULS-SGGW), Nowoursynowska 159, Warsaw, 02-776 Poland

**Keywords:** Antioxidants, Cytokinins, In vitro plant culture, Kale, Micropropagation, Sterols

## Abstract

**Background:**

Kale, a versatile cruciferous crop, valued for its pro-health benefits, stress resistance, and potential applications in forage and cosmetics, holds promise for further enhancement of its bioactive compounds through in vitro cultivation methods. Micropropagation techniques use cytokinins (CKs) which are characterized by various proliferative efficiency. Despite the extensive knowledge regarding CKs, there remains a gap in understanding their role in the physiological mechanisms. That is why, here we investigated the effects of three CKs – kinetin (Kin), 6-benzylaminopurine (BAP), and 2-isopentenyladenine (2iP) – on kale physiology, antioxidant status, steroidal metabolism, and membrane integrity under in vitro cultivation.

**Results:**

Our study revealed that while BAP and 2iP stimulated shoot proliferation, they concurrently diminished pigment levels and photosynthetic efficiency. Heightened metabolic activity in response to all CKs was reflected by increased respiratory rate. Despite the differential burst of ROS, the antioxidant properties of kale were associated with the upregulation of guaiacol peroxidase and the scavenging properties of ascorbate rather than glutathione. Notably, CKs fostered the synthesis of sterols, particularly sitosterol, pivotal for cell proliferation and structure of membranes which are strongly disrupted under the action of BAP and 2iP possibly via pathway related to phospholipase D and lipoxygenase which were upregulated. Intriguingly, both CKs treatment spurred the accumulation of sitostenone, known for its ROS scavenging and therapeutic potential. The differential effects of CKs on brassicasterol levels and brassinosteroid (BRs) receptor suggest potential interactions between CKs and BRs.

**Conclusion:**

Based on the presented results we conclude that the effect evoked by BAP and 2iP in vitro can improve the industrial significance of kale because this treatment makes possible to control proliferation and/or biosynthesis routes of valuable beneficial compounds. Our work offers significant insights into the nuanced effects of CKs on kale physiology and metabolism, illuminating potential avenues for their application in plant biotechnology and medicinal research.

## Background

Kale (*Brassica oleracea* convar. *acephala* var. *sabellica*) stands as one of the most crucial cruciferous crops, representing the oldest variety within its species. This species is renowned for its robust pro-health attributes and remarkable resilience to abiotic stresses, particularly due to its antioxidant properties [1]. Beyond agricultural significance, kale emerges as a versatile forage material with the potential to enhance egg production and qualities, boasting elevated content of lutein and n-3 fatty acids [2]. The multifaceted benefits of kale extend to its recognized anticancerogenic effect attributed to antigenotoxic extract properties, notably enriched with sulforaphane [3, 4]. Moreover, kale harbors the potential to contribute to the cosmetics industry, with its bounty of flavonoids, including kaempferol and quercetin derivatives, offering possible applications in skincare formulations [5].

In vitro techniques play a pivotal role in supporting both conventional and molecular breeding programs, offering a suite of advantages including disease elimination, selection of novel, elite lines, and rapid multiplication. Tissue cultures hold the potential to outpace traditional agriculture [6]. Within these cultures, explants undergo dynamic changes in the concentration and transport direction of trophic substances and plant growth regulators (PGRs) uptaken also from the growth medium. This environment exposes plant tissues to a range of phytohormones, with cytokinins (CKs) emerging as key players due to their capacity to efficiently stimulate shoot proliferation or single-cell growth [7, 8].

CKs, like other plant hormones, exhibit diverse biological effects [9], exerting a profound influence on numerous aspects of plant growth and development. There are two groups of adenine-derived CKs of natural origin: more commonly for plants - isoprenoid CKs (e.g. N^6^-(2-isopentenyl)adenine, 2iP) and aromatic (e.g. kinetin, Kin; 6-benzylaminopurine, BAP). In general, both groups exhibit similar activity but they may differ in various processes [10]. The regulatory role of CKs in ontogenesis primarily revolves around modulating cell proliferation and differentiation, thereby fostering the robust growth of above-ground plant structures by activating apical and lateral meristems as well as cambia. It has been proposed that biotechnological adjustments in CKs content could bolster plant survival and productivity in challenging environmental conditions [10, 11]. Studies on root hairs of *Medicago sativa* have revealed that extracellular CKs prompt alterations in membrane transport, leading to membrane hyperpolarization. Furthermore, different CKs elicit varying effects on membrane potential [12] and engage in a range of signaling pathways [9]. This intricate interplay underscores the versatile role of CKs in regulating cellular processes and emphasizes their significance in both plant signaling and development.

Cell membranes serve as crucial receptors for various environmental signals. When exposed to stress, these membranes undergo significant alterations, particularly in the compositions of sterols – vital compounds responsible for membrane permeability, fluidity, transmission of stress signals, the function of membrane-associated enzymes, and proton pumps [13]. Sterol precursors are synthesized via the cytoplasmic mevalonate pathway (MVA), wherein 3-hydroxy-3-methylglutaryl-CoA reductase (HMGR) serves as a pivotal enzyme [14]. Notably, HMGR inhibitors include a range of low-molecular-weight compounds such as ascorbate, dehydroascorbate, and glutathione, which are integral to the Foyer-Halliwell-Asada cycle [15, 16]. These compounds might be overaccumulated in plants obtained under in vitro conditions [17]. Indeed, plant cell and tissue cultures are well-documented to synthesize and accumulate valuable secondary metabolites, including antioxidants, under in vitro conditions, potentially indicating increased antioxidant enzyme activity [18].

**Fig. 1:**
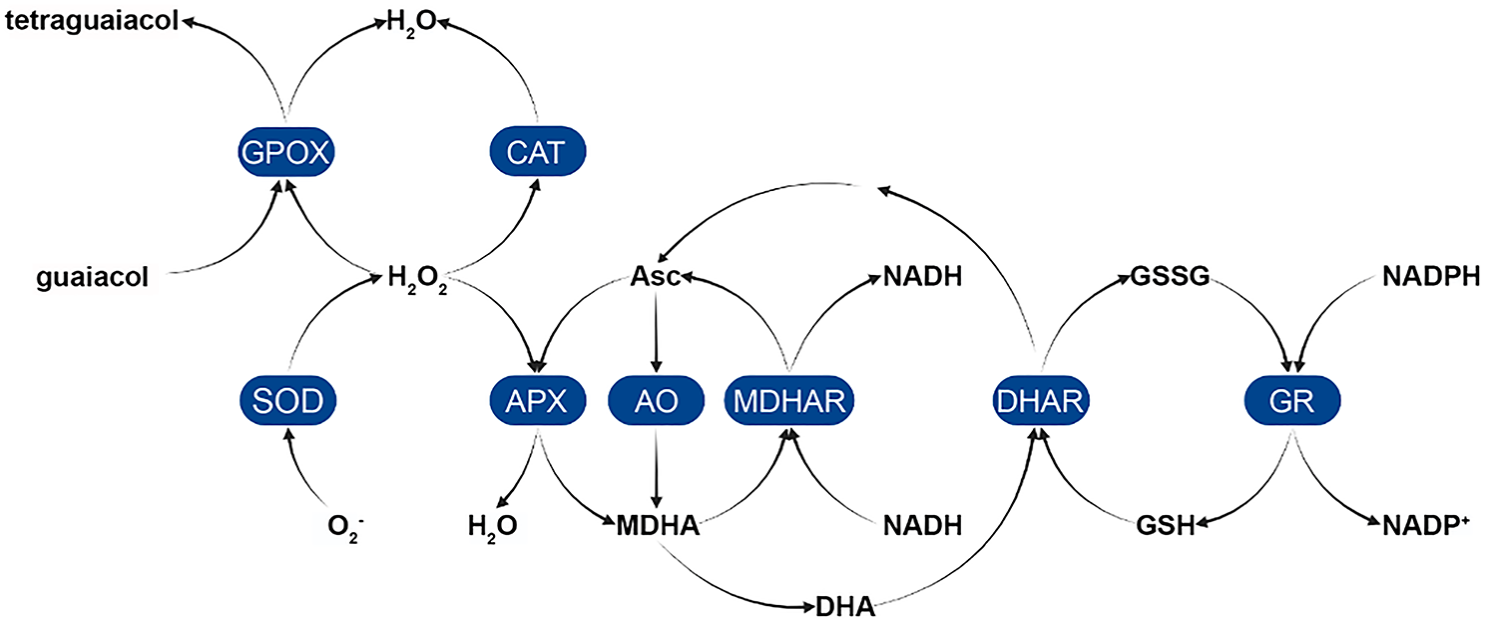
Enzymatic antioxidant system in plants including the Foyer-Halliwell-Asada cycle elements. Enzymes: GPOX - guaiacol peroxidase; CAT – catalase; SOD – superoxide dismutase; APX - ascorbate peroxidase; AO – ascorbate oxidase, MDHAR – monodehydroascorbate reductase; DHAR – dehydroascorbate reductase; and GR – glutathione reductase. Non-enzymatic compounds: Asc – ascorbic acid; MDHA – monodehydroascorbate; DHA – dehydroascorbic acid; GSSG – glutathione disulfide; GSH – glutathione

The antioxidant status relies on a multitude of enzymes and endogenous low-molecular-weight antioxidants (Fig. 1; [18]). Among these, the concentration of reactive oxygen species (ROS) is intricately regulated by a cascade of redox pairs: reduced glutathione (GSH) – glutathione disulfide (GSSG) and reduced ascorbate (Asc) – dehydroascorbate (DHA), which are maintained in balance by NADP^+^ and NADPH [19]. The oxidation of Asc and GSH occurs in response to the presence of ROS, such as superoxide radical (O_2_^•-^) [15]. Alterations in the ratio and level of reduced and oxidized forms can significantly impact cellular redox buffering capacity. Additionally, the ascorbate-glutathione pathway stands out as pivotal in H_2_O_2_ metabolism [19].

We have shown previously that various CKs evoked differential responses of kale under in vitro and are not dependent on their structure, since BAP and 2iP caused shoot proliferation, while Kin has not evoked such effect [20]. Remarkably, we achieved successful micropropagation of *B. oleracea* varieties, which are considered challenging for such techniques. Despite the extensive knowledge regarding CKs, there remains a gap in understanding their role in the physiological mechanisms, particularly under specific conditions - in vitro cultivation in crop species such as kale. Significant questions are: (1) how particular CKs influence basic metabolic pathways including respiration; (2) do they interact with other phytohormones, e.g. BRs; (3) can they change membrane functioning and evoke the production of beneficial compounds, e.g. antioxidants and sterols? Therefore, in this study, we performed comprehensive analyses using physiological, biochemical, and instrumental methods to answer these questions. Future studies delving into the intricate interplay between CKs and other signaling pathways switched under in vitro culture will be crucial for harnessing their full therapeutic and biotechnological potential.

## Materials and methods

### Plant material, kale treatments with CKs and cultivation

To test the impact of various CKs on different parameters of kale growth we cultivate plants under in vitro conditions. The initiation of adventitious shoots culture was conducted using previously published protocol [20]. Stock of each cytokinin was prepared using 1 N HCl (2iP, Kin) or 1 N KOH (BAP). Shoot tips were isolated from 2-week-old seedlings and placed in glass jars (540 mL) containing 60 mL solidified with 0.8% Plant Agar (Duchefa Biochemie, The Netherlands) Murashige and Skoog (MS) medium including vitamins (Duchefa Biochemie, The Netherlands) supplemented with 3% sucrose and 2.5 mg/dm^3^ CKs (2iP, BAP or Kin; Sigma-Aldrich, USA). Control plants were transferred onto fresh MS medium without any CK. Eight explants were transferred to each jar. Culture was maintained at 22 ± 1 °C under 14/10 photoperiod with a quantum irradiation intensity of 100 µmol·m^-2^·s^-1^ photosynthetically active radiation (PAR). All biochemical analyzes were performed using leaves of kale after the first four weeks of culture. Fresh material was used for analyses of chlorophyll a fluorescence, respiration rate, enzymes activity, and electrical conductivity (EC) test. Collected tissues were air-dried for sterol determination, while for the rest of assays, kale leaves were frozen in liquid nitrogen and stored under − 80 °C.

### Pigment content

Photosynthetic pigments were calculated from absorbances using equations proposed by Lichtenthaler and Buschmann [21]. In brief, 0.1 g of leaves were homogenized in 1.5 ml ethanol (EtOH)/acetone (1:1, v/v) and 0.02% butylated hydroxytoluene. The obtained extracts were centrifuged at 12,000 rpm for 5 min and the absorbance (at 649 nm and 664 nm for chlorophylls, while 470 nm for carotenoids) was measured with a 1 cm optical path length.

### Chlorophyll a fluorescence

Chlorophyll a fluorescence was measured after 30 min of dark adaptation using a portable chlorophyll fluorometer (Handy-PEA, Hansatech Instruments, UK). The maximum saturating flash was 3000 µmol/m/s and chlorophyll a emission was induced by 1 s pulse. Photosystem II photochemical potential quantum efficiency (the ratio of variable to maximum fluorescence - Fv/Fm) was recorded.

### Respiration rate

Infrared absorption properties of CO_2_ were used to measure R_dark_ (as respiratory CO_2_ efflux) with an infrared gas analyzer (Q-S151, Qubit Systems). Seedlings were adapted to dark for 30 min and then transferred from glass jars to the dark measuring chamber (of volume 10 ml). Measurements have been started once CO_2_ readings stabilized (~ 10 min) for 5 min. The flow was 244 ml/min.

### NAD/NADH and NADP/NADPH determination

The content of nicotinamide adenine dinucleotides was determined using enzyme cycling assays, employing 3-(4,5-dimethylthiazolyl-2)-2,5-diphenyltetrazolium bromide (MTT) as the terminal electron acceptor, with phenazine methosulfate (PMS) serving as an electron carrier. The reaction was based on either the conversion of EtOH to acetaldehyde, catalyzed by alcohol dehydrogenase (ADH, EC 1.1.1.1) for NAD(H) determination, or the conversion of glucose-6-phosphate (G6P) to 6-phosphogluconolactone by glucose-6-phosphate dehydrogenase (G6PDH, EC 1.1.1.49) for NADP(H) determination [22, 23].

Kale leaves (250 mg) were homogenized in 1 mL of 0.1 M HCl (for NAD or NADP determination) or in 1 mL of 0.1 M NaOH (for NADH or NADPH determination), heated at 100 °C for 5 min, cooled, centrifuged (10,000 rpm for 10 min at 4 °C), and neutralized with NaOH or HCl, respectively. The reaction mixture contained equal volumes of 0.1 M bicine buffer, 40 mM EDTA, 4.2 mM MTT, 16.6 mM PMS, and substrates (5 M EtOH or 25 mM G6P). After preheating at 37 °C for 5 min of the reaction mixture (500 µL) with 0.1 M NaCl (350 µL) and plant extract (50 µL), the enzyme reaction was initiated by adding 100 µL of 100 U/ml ADH (Merck, Germany) for NAD(H) determination, or 100 µL of 14 U/ml G6PDH (Merck, Germany) for NADP(H) determination. Reactions lasted 40 min at 37 °C and were stopped by adding 6 M NaCl (500 µL). After centrifugation at 16,000 rpm for 10 min at 4 °C, the obtained pellet was solubilized in 1 mL of 96% EtOH and measured at 570 nm. The contents of nicotinamide adenine dinucleotides were expressed in nmol/g FW.

### Determination of O_2_^•-^ and H_2_O_2_ level

The quantification of O_2_^•-^ content followed the methodology outlined by Han et al. [24]. This involved a reaction of 500 µL supernatant (600 mg of kale leaves were homogenized in 100 mM sodium phosphate buffer (NaP) pH 7.8 supplemented with 0.1% polyvinylpyrrolidone (PVP) with 1 mM hydroxylamine hydrochloride (500 µL), 17 mM p-aminobenzenesulfonic acid (500 µL) and 7 mM α-naphthylamine (500 µL). Absorbance was measured at 530 nm, and O_2_^•-^ content was expressed as nmol/g FW. A standard curve with NO_2_^-^ was utilized for quantification [25]. For the extraction and quantification of H_2_O_2_, the method described by Junglee et al. [26] was employed with slight modifications. Fresh leaf samples (100 mg) were homogenized in 1% (w/v) trichloroacetic acid (TCA). Following centrifugation (13,000 rpm, 15 min, 4 °C), supernatants were mixed with 10 mM K-phosphate buffer pH 7 and 1 M potassium iodide (KI), then incubated in the dark for 15 min. Absorbance was recorded at 350 nm. H_2_O_2_ content in samples was calculated using a calibration curve.

### Antioxidant enzyme extractions and assays

The activity of three antioxidant enzymes, catalase (CAT), superoxide dismutase (SOD), and guaiacol peroxidase (GPOX), was assayed spectrophotometrically.

Leaves of kale were homogenized in suitable sodium phosphate buffer (NaP): 50 mM NaP with 1 mM EDTA, 1% PVP, 1 M NaCl (for CAT), and 1 mM Asc (for SOD), or 15 mM NaP (for GPOX) on ice. Samples were then centrifuged (17,000 rpm for 15 min at 4 °C), and the supernatant was used for determining enzyme activity. CAT (EC 1.11.1.6) activity was assayed by measuring the decomposition of H_2_O_2_ at 240 nm, according to Dhindsa et al. [27]. The reaction mixture contained 50 mM NaP buffer (pH 7.0) (1825 µL), plant extract (25 µL), and 1% H_2_O_2_ solution (150 µL). SOD (EC 1.15.1.1) activity was determined according to the method of Beauchamp and Fridovich [28], based on the inhibition of photochemical reduction of NBT [29]. For determination 50 µL of supernatant was mixed with 2.3% riboflavin (100 µL) and mixture (2850 µL) prepared by dissolving 2.9 mg EDTA, 6 mg NBT and 194 mg methionine in 100 ml 50 mM NaP buffer (pH 6.4). Reaction mixture was placed in 50 mL beaker and exposed to UV lights for 10 min. Absorbance was measured before and after UV treatment. GPOX (EC 1.11.1.7) activity was assessed by examining the conversion of 0.1 mM guaiacol in the presence of 1 mM H_2_O_2_, with the progress monitored through the formation of tetraguaiacol observed at 470 nm. The assay mixture consisted of 15 mM NaP buffer (pH 6.0) and enzyme extract in a total volume of 500 µl, and 1500 µL of substrate consisting of the aforementioned components [30]. The activities of CAT and GPOX were expressed as millimole per minute per milligram protein (mmol/min/mg protein). For SOD one unit of activity was defined as a 50% decrease in SOD-NBT reduction. Protein content was measured according to the method of Lowry et al. [31] with bovine serum albumin (BSA) used as a standard.

### Functioning of ascorbate – glutathione cycle

The determination of APX (EC 1.11.1.11) activity was performed based on the H_2_O_2_-dependent oxidation of Asc to DHA, analyzed at 265 nm. For extraction, 50 mM NaP (pH 7.0) buffer with the addition of 1 mM EDTA, 1% PVP, 1 M NaCl, and 1 mM Asc was used. The reaction mixture comprised 50 mM NaP (7.0) (1800 µL), 1 mM EDTA (25 µL), 5 mM Asc (50 µL), 1 mM H_2_O_2_(100 µL), and kale homogenate (25 µL) [32]. AO (EC 1.10.3.3) activity was determined from the absorbance decrease at 265 nm of the reaction mixture containing 60 mM NaP (pH 6.1) with 0.5 mM EDTA (1880 µL), 2 mM Asc (100 µL), and kale extract (10 µL). For enzyme extraction 60 mM NaP (pH 6.1) with 0.5 mM EDTA was used [33]. MDHAR (EC 1.6.5.4) activity was assayed as the rate of monodehydroascorbate-dependent oxidation of NADPH according to De Gara et al. [34]. The supernatant (20 µL) was tested by measuring the oxidation rate of NADH at 340 nm in a reaction mixture composed of 50 mM Tris-HCl (pH 8.0) (780 µL), 0.2 mM NADH (200 µL), 1 mM Asc (1 mL), and 0.2 units of ascorbate oxidase from Cucurbita sp. (Merck, Germany). DHAR (EC 1.8.5.1) activity was determined as the rate of glutathione-dependent formation of Asc. Extraction was performed in 50 mM NaP (pH 6.8). The reaction mixture contained the same buffer (1580 µL), 2 mM GSH (200 µL), 1 mM DHA (200 µL), and homogenate (20 µL) [35]. GR (ED 1.8.1.7) activity was determined by following glutathione disulfide-dependent oxidation of NADPH. Enzyme was isolated in 100 mM NaP (pH 7.8) supplemented with 2 mM EDTA and 1% PVP. The decrease of absorbance was measured for a mixture of 100 mM NaP with 2 mM EDTA (2 mL), 0.2 mM NADPH (30 µL), 0.5 mM GSSG (100 µL), and plant extract (100 µL) [36]. The activity of all enzymes was expressed as mmol/min/mg protein.

The assay for glutathione determination is based on the reaction of GSH with 5,5′-dithio-bis(2-nitrobenzoic acid) (DTNB) as described by Rahman et al. [37]. Frozen samples (50 mg) were ground with 1% sulfosalicylic acid (1.5 mL) and centrifuged (17,000 rpm for 15 min at 4 °C). For GSSG determination, the supernatant (300 µL) was mixed with 2-vinylpyridine (6 µL), while for GSH + GSSG, it was mixed with sulfosalicylic acid (6 µL). After 1 h incubation at RT, 1 M triethanolamine (18 µL) was added for a further 10 min. The reaction mixture contained 0.1 M potassium phosphate buffer with 5 mM EDTA (pH 7.5) (600 µL), a sample (100 µL), 0.3 mM DTNB (60 µL), 1.2 U glutathione reductase (GR; Merck, Germany), and 0.2 mM NADPH (60 µL). Absorbance was recorded for 4 min at 412 nm [37].

The determination of Asc involved the reduction of Fe^3+^ to Fe^2+^ with Asc, followed by the complexation of the reduced iron with α,α’-dipyridyl. Simultaneously, DHA was quantified in the same samples. While DHA itself does not contribute to the formation of the mentioned complex, the addition of dithiothreitol (DTT) as a reducing agent for DHA indirectly facilitates the assessment of the total content of both Asc and DHA [38]. Ground kale leaves (200 mg) underwent extraction with 5% TCA (750 µL) and were subsequently centrifuged (10,000 rpm for 10 min at 4 °C). For Asc determination, the supernatant (270 µL) was mixed with H_2_O (67 µL), while the mixture for Asc + DHA included 10 mM DTT (33.5 µL) and 80 mM K_2_HPO_4_ (33.5 µL). In both cases, 85% H_3_PO_4_ (80 µL), 0.5% α,α’-dipyridyl (1370 µL), and 1% FeCl_3_ (280 µL ) were added. After a one-hour incubation at RT, absorbance was recorded at 525 nm.

### Cell membrane modifications

To assess the impact of CKs on membrane functioning, we evaluated the leakage of exudates. Fresh leaves (1 g) were thoroughly washed with deionized water to remove absorbed ions. Subsequently, the tissues were immersed in 10 mL of deionized water for 1 h in the darkness, and the EC was measured. Following this, the leaves were boiled at 90 °C, vigorously shaken, allowed to cool to room temperature, and EC was measured again. Membrane destabilization [%] was then calculated based on the EC values obtained from samples exhibiting total leakage.

The level of MDA, a marker for lipid peroxidation, was determined following the method described by Hodges et al. [39]. Plant tissue was homogenized in a ratio of 1:25 (g fresh weight: ml) 80% (v/v) EtOH. The resulting extracts were then centrifuged (12,000 rpm, 10 min, 4 °C), and 0.5 ml of supernatants were mixed with an equal volume of either – thiobarbituric acid (TBA) solution containing 20% (w/v) TCA and 0.01% butylated hydroxytoluene or + TBA solution containing 20% (w/v) TCA, 0.01% butylated hydroxytoluene, and 0.65% TBA. The mixtures were incubated at 95 °C for 25 min, followed by centrifugation (12,000 rpm, 10 min, 4 °C). Absorbance readings were taken at 440 nm, 532 nm, and 600 nm and used for subsequent calculations.

### Dot blot analyses

Frozen material (0.2 g) was homogenized in liquid nitrogen, and the resulting powder was mixed for 5 min with 500 µL of extraction buffer (50 mM TRIS-HCl pH 6.8, 1% SDS, 5% glycerol, 2.5 mM EDTA, 1 mM DTT), followed by incubation for 30 min at 4 °C. Protein suspensions were clarified by centrifugation (13,000 rpm, 15 min, 4 °C), and the obtained supernatants were used for protein quantification following Bradford’s procedure [40]. Crude extracts were used for dot blot analysis, where 100 ng of proteins were loaded onto nitrocellulose membranes (Protran BA 83, Whatman GmBH). Non-specific sites of binding were blocked with 1% non-fat milk in TBS pH 7.5 for 30 min at RT. Subsequently, membranes were incubated with primary antibodies detected PHOSPHOLIPASE D (anti-PLD, AS09 556), LIPOXYGENASE (anti-LOX, AS06 128), BRASSINOSTEROID INSENSITIVE1 (anti-BRI1, AS12 1859) provided by Agrisera (Sweden) at a dilution of 1:1000 in TBS pH 7.5. Afterward, membranes were washed 3 times for 5 min with TBS pH 7.5 and probed with DyLight 488-conjugated anti-rabbit IgG secondary antibody (AS09 633) diluted 1:2000 in TBS pH 7.5. After a 2 h incubation in the darkness at RT, membranes were washed three times for 5 min with TBS pH 7.5. The signal was visualized using a ChemiDocTM Touch Imaging System. For all detected spots, image processing software (ImageJ) was used for densitometry measurements, and average values were presented on charts.

### Steroids metabolism

The activity of HMGR was assessed using the protocol outlined by Kumar et al. [41]. The method involves quantifying the reduction in absorbance at 340 nm, which is indicative of NADPH oxidation catalyzed by HMGR in the presence of HMG-CoA as a substrate. The resulting HMGR activity was expressed as µmol NADPH/min/mg protein.

For the quantification and qualification of steroids, dried kale samples (300 ± 50 mg) were initially ground into powder and subjected to extraction using a Soxhlet apparatus. The extraction process involved an 8 h cycle with diethyl ether to extract fractions of free and conjugated (esters and low-polar glycosides) sterols, followed by another 8 h cycle with methanol to extract more polar sterol glycosides. The resulting extracts were then evaporated under reduced pressure and underwent fractionation by TLC over silica gel 60 H (Merck, Darmstadt, Germany) using a chloroform: methanol 97:3 (v/v) mixture as the eluent. This process yielded three distinct fractions: esters, free steroids, and glycosides. The fraction containing free steroids was directly analyzed using a gas chromatography-mass spectrometer (GC-MS; Agilent Technologies 7890 A) equipped with a 5975 C mass spectrometric detector. Meanwhile, the ester and glycoside fractions underwent alkaline and acidic hydrolysis, respectively, before being subjected to GC-MS analysis. Samples were dissolved in a mixture of diethyl ether and methanol (5:1, v/v) and applied (in a volume of 1–4 µl) using a 1:10 split injection. The Ultra-Inert GC column HP-5MS (30 m x 0.25 mm i.d. film thickness 0.25 μm) (Agilent Technologies, Santa Clara, CA) was used. Helium served as the carrier gas at a flow rate of 1 ml/min. Separation was conducted with temperature programming: an initial temperature of 160 °C held for 2 min, then increased to 280 °C at 5 °C/ min, and a final temperature of 280 °C held for 44 min. Other parameters included: inlet and flame ionization detector (FID) temperature at 290 °C, MS transfer line temperature at 275 °C, quadrupole temperature at 150 °C, ion source temperature at 230 °C, EI at 70 eV, m/z range of 33–500; FID gas (H_2_) flow at 30 mL/min (from a hydrogen generator), and air flow at 400 mL/min. Individual compounds were identified by comparing their mass spectra with library data from Wiley 9^th^ Ed. and NIST 2008 Lib. SW Version 2010, or previously reported data, and by comparison of their retention times and corresponding mass spectra with those of standards (cholesterol, campesterol, brassicasterol, sitosterol, stigmasterol). Quantitation was done using an external standard method based on calibration curve determined for sitosterol [42].

### Statistical analysis

For micropropagation eight explants per jar were used for each treatment and the experiments were repeated three times. Each analysis was performed in five biological replications. Data were evaluated by ANOVA followed by post–hoc Tukey’s test (*p*-value of less than 0.05 considered statistically significant) in Statistica ver.14 (StatSoft, Inc). The bars in the graphs represent the mean ± the standard deviation.

## Results

### Physiological response to treatment with exogenous cytokinins

**Fig. 2:**
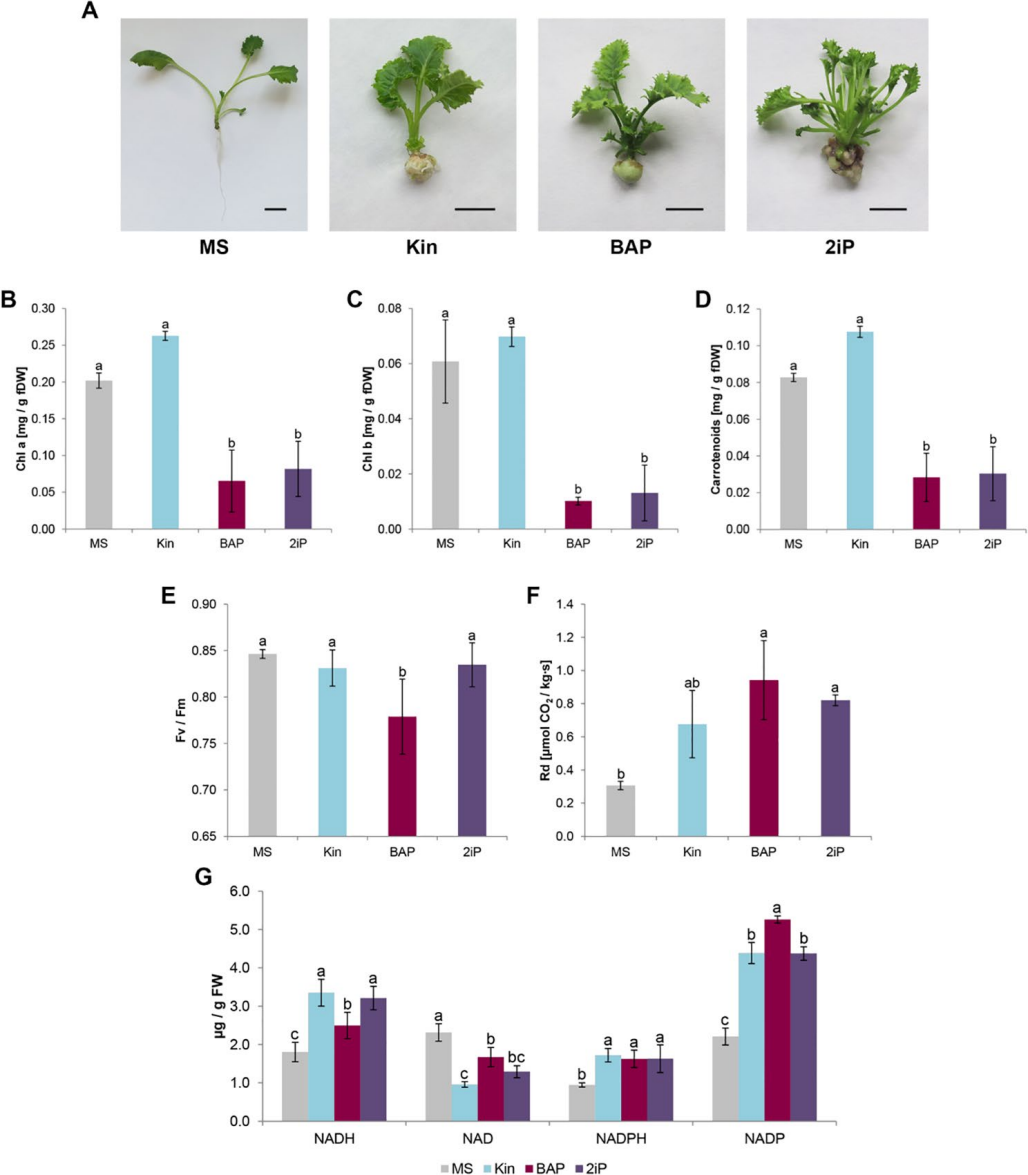
The response of kale on different cytokinins under in vitro conditions. Kale microshoots after 4 weeks on MS medium without phytohormones and supplemented with different cytokinin at a concentration of 2.5 mg/dm^3^: kinetin (Kin), 6-benzylaminopurine (BAP), N^6^-(2-isopentenyl)adenine (2iP) (**A**). Bar = 1 cm. Content of chlorophyll a (**B**), chlorophyll b (**C**), and carotenoids (**D**), maximum quantum yield of photosystem II (Fv/Fm; **E**), respiration rate (**F**), and nicotinamide adenine dinucleotide (NAD/NADH, NADP/NADPH; **G**) level in kale leaves under in vitro conditions. Different letters imply significant differences (*p* ≤ 0.05, Tukey test)

The most significant proliferative effect was observed with BAP treatment. Plants cultivated under in vitro conditions in the presence of either 2iP or BAP developed well, and have many small leaves in comparison to the control growing on MS medium. However, Kin has not evoked such effect, since these microplantlets resembled the control. While treatment with 2iP stimulated proliferation, it was not as pronounced as with BAP. Interestingly, leaves treated with 2iP exhibited a distinct morphology, appearing more ruffled in shape compared to others. Additionally, control plants developed long roots not observed under CK treatment (Fig. 2A).

Given the various color of kale microshoots cultivated in the presence of different CKs, in the next step we analyze the pigment concentration in the leaves. Surprisingly, both BAP and 2iP treatments had a negative impact on the levels of chl a, chl b, and carotenoids (Fig. 2B-D). However, the concentrations of these compounds were comparable between plants treated with BAP or 2iP. Conversely, Kin treatment did not significantly alter the accumulation of chl a and carotenoids when compared to the control.

Among all the treated plants, only the application of BAP led to changes in the photosystem II photochemical potential quantum efficiency (Fv/Fm parameter). In control, Fv/Fm was 0.846 ± 0.005, while BAP application resulted in a decrease of this value to 0.779 ± 0.040 (Fig. 2E). Irrespective of the CK used, the respiratory rate was higher compared to control plants. The highest value was observed following BAP treatment (0.94 ± 0.24 µmol CO_2_/kg‧s). After the application of Kin or 2iP, the respiration rate was almost double (0.68 ± 0.20 and 0.82 ± 0.03 µmol CO_2_/kg‧s, respectively) compared to MS cultivated plants (0.31 ± 0.03 µmol CO_2_/kg‧s) (Fig. 2F).

The addition of CK to the growing medium significantly stimulated the reduction of NAD, as evidenced by the increased level of NADH and decreased level of NAD compared to the control (Fig. 2G). This correlation was particularly evident for shoots treated with Kin, where the level of NADH was 3.35 ± 0.35 µg/g FW and NAD were 0.96 ± 0.07 µg/g FW. In contrast, the derivative NADP was observed to be predominantly in its oxidized form in the presence of CKs, especially after BAP treatment, where the level of NADPH was 1.63 ± 0.23 µg/g FW and NADP was 5.26 ± 0.09 µg/g FW.

### Modulation of oxidative status by cytokinins

**Fig. 3:**
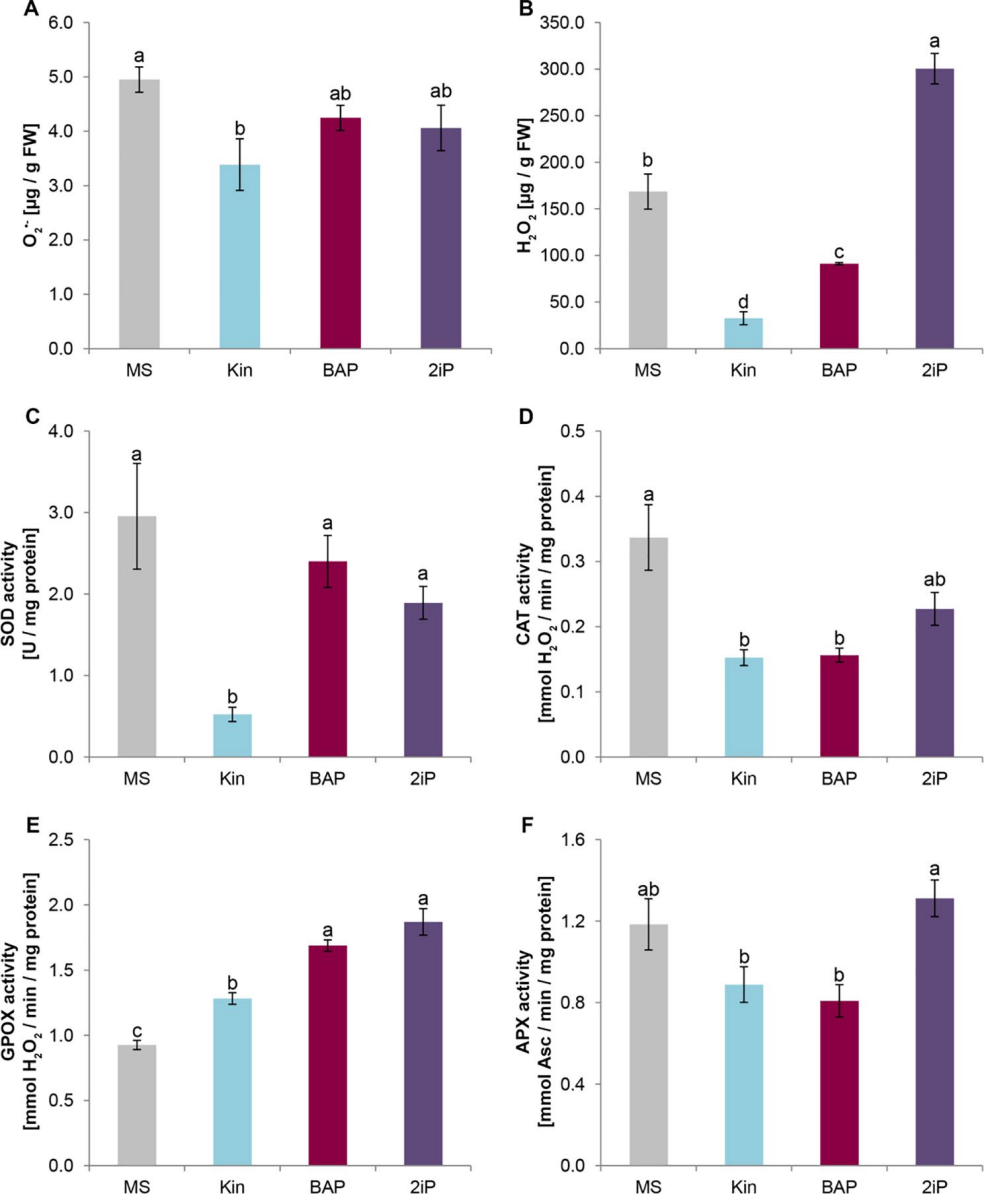
The impact of cytokinins on ROS content and activity of particular elements of antioxidant system in kale under in vitro conditions. Kale microshoots were grown on MS medium without phytohormones and supplemented with different cytokinin at a concentration of 2.5 mg/dm^3^: kinetin (Kin), 6-benzylaminopurine (BAP), N^6^-(2-isopentenyl)adenine (2iP). The content of ROS: O_2_^•-^ (**A**) and H_2_O_2_ (**B**), activity of superoxide dismutase (SOD; **C**), catalase (CAT; **D**), guaiacol peroxidase (GPOX; **E**), and ascorbate peroxidase (APX; **F**). Different letters imply significant differences (*p* ≤ 0.05, Tukey test)

Regarding the accumulation of the O_2_^•-^, Kin exhibited the capacity to suppress its levels by over 30% (3.39 ± 0.47 µg/g FW compared to the control’s 4.95 ± 0.23 µg/g FW). Neither BAP nor 2iP significantly affected the accumulation of the O_2_^•-^ (Fig. 3A). Aromatic CKs (Kin and BAP) demonstrated a notable reduction in H_2_O_2_ levels compared to control plants. Conversely, 2iP stimulated the production of H_2_O_2_ (300.5 ± 16.3 µmol/g FW), reaching levels exceeding twice that of the control (168.5 ± 18.9 µmol/g FW) (Fig. 3B).

The activity of SOD was found to be highest in control plants cultivated on MS medium (2.96 ± 0.65 U/mg protein; Fig. 3C). A slightly lower activity was observed for 2iP and BAP, though this difference did not achieve statistical significance. Notably, plants treated with Kin exhibited the lowest SOD activity (0.52 ± 0.09 U/mg protein). Control plants also showed the highest CAT activity (0.34 mmol H_2_O_2_/min/mg protein; Fig. 3D), but conversely, the lowest GPOX activity (0.93 mmol H_2_O_2_/min/mg protein; Fig. 3E). Interestingly, under CK treatment, the highest activity for both enzymes was observed in plants propagated on a medium containing 2iP (CAT 0.23 mmol H_2_O_2_/min/mg protein; GPOX 1.87 mmol H_2_O_2_/min/mg protein). None of the CKs affected the activity of APX (Fig. 3F).

### Functioning of ascorbate – glutathione cycle

**Fig. 4:**
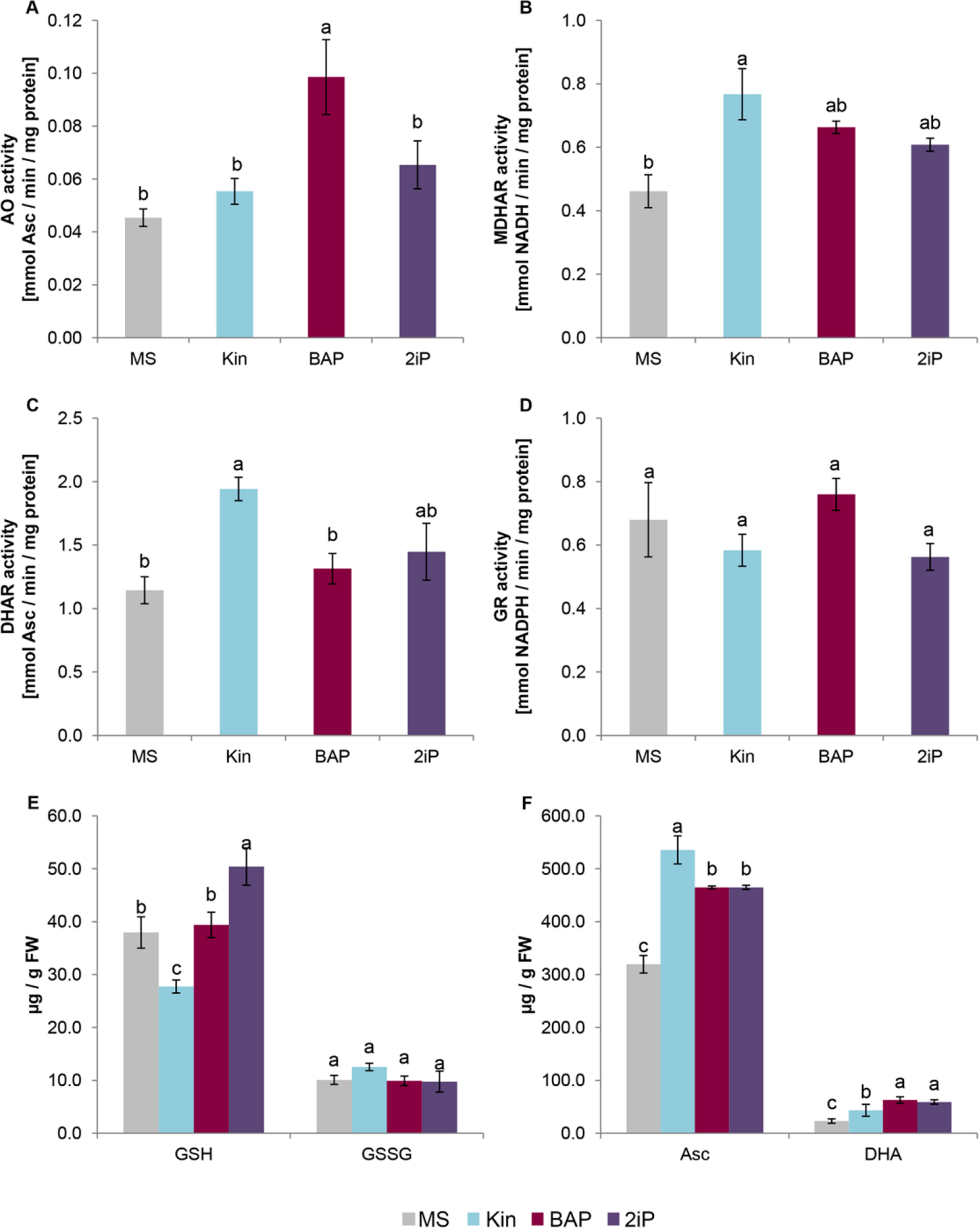
The functioning of Foyer-Halliwell-Asada pathway in kale leaves under various cytokinins treatment. Kale microshoots were grown on MS medium without phytohormones and supplemented with different cytokinin at a concentration of 2.5 mg/dm^3^: kinetin (Kin), 6-benzylaminopurine (BAP), N^6^-(2-isopentenyl)adenine (2iP). The activity of ascorbate oxidase (AO; **A**), monodehydroascorbate reductase (MDHAR; **B**), dehydroascorbate reductase (DHAR; **C**), and glutathione reductase (GR; **D**) and the content of glutathione (**E**) and ascorbate (**F**) in both reduced and oxidized forms in kale microshoots. Different letters imply significant differences (*p* ≤ 0.05, Tukey test)

Among the enzymes involved in the Foyer-Halliwell-Asada pathway, CKs significantly affected the activity of AO, MDHAR, and DHAR (Fig. 4A-C). Treatment with BAP exhibited a more than twofold increase in AO activity, rising to 0.099 ± 0.014 mmol Asc/min/mg protein compared to the control plants with AO activity of 0.045 ± 0.003 mmol Asc/min/mg protein (Fig. 4A). On the other hand, Kin increased the activity of enzymes involved in the Asc regeneration, such as MDHAR (Fig. 4B) and DHAR (Fig. 4C). Shoots treated with this CK displayed MDHAR activity at 0.767 ± 0.081 mmol NADH/min/mg protein (compared to 0.462 ± 0.052 mmol NADH/min/mg protein in the control) and DHAR activity at 1.942 ± 0.092 mmol Asc/min/mg protein (in contrast to 1.144 ± 0.106 mmol Asc/min/mg protein in the control). For GR no effect of CK was observed when compared to the control kale (Fig. 4D).

The analysis of obtained kale shoots included also an examination of their reduced and oxidized ascorbate and glutathione contents (Fig. 4E and F). The reduced forms of both compounds were found to be several times higher than their respective oxidized forms. The presence of CK in the medium significantly influenced the accumulation of GSH. Specifically, Kin decreased the content of GSH (27.7 ± 1.2 µg/g FW), while 2iP caused its accumulation (50.4 ± 3.5 µg/g FW). No significant effect on the level of GSH, compared to the control, was observed for BAP, and there was no significant impact on the level of GSSG for any CK. In contrast, the levels of Asc and its oxidized form DHA were significantly higher following CK treatment compared to the control. The most robust stimulation of Asc accumulation was noted for Kin (535.6 ± 26.4 µg/g FW), while both BAP and 2iP treatment resulted in DHA increase (464.9 ± 24.6 µg/g FW and 464.9 ± 3.9 µg/g FW, respectively).

### Cell membrane modification

**Fig. 5:**
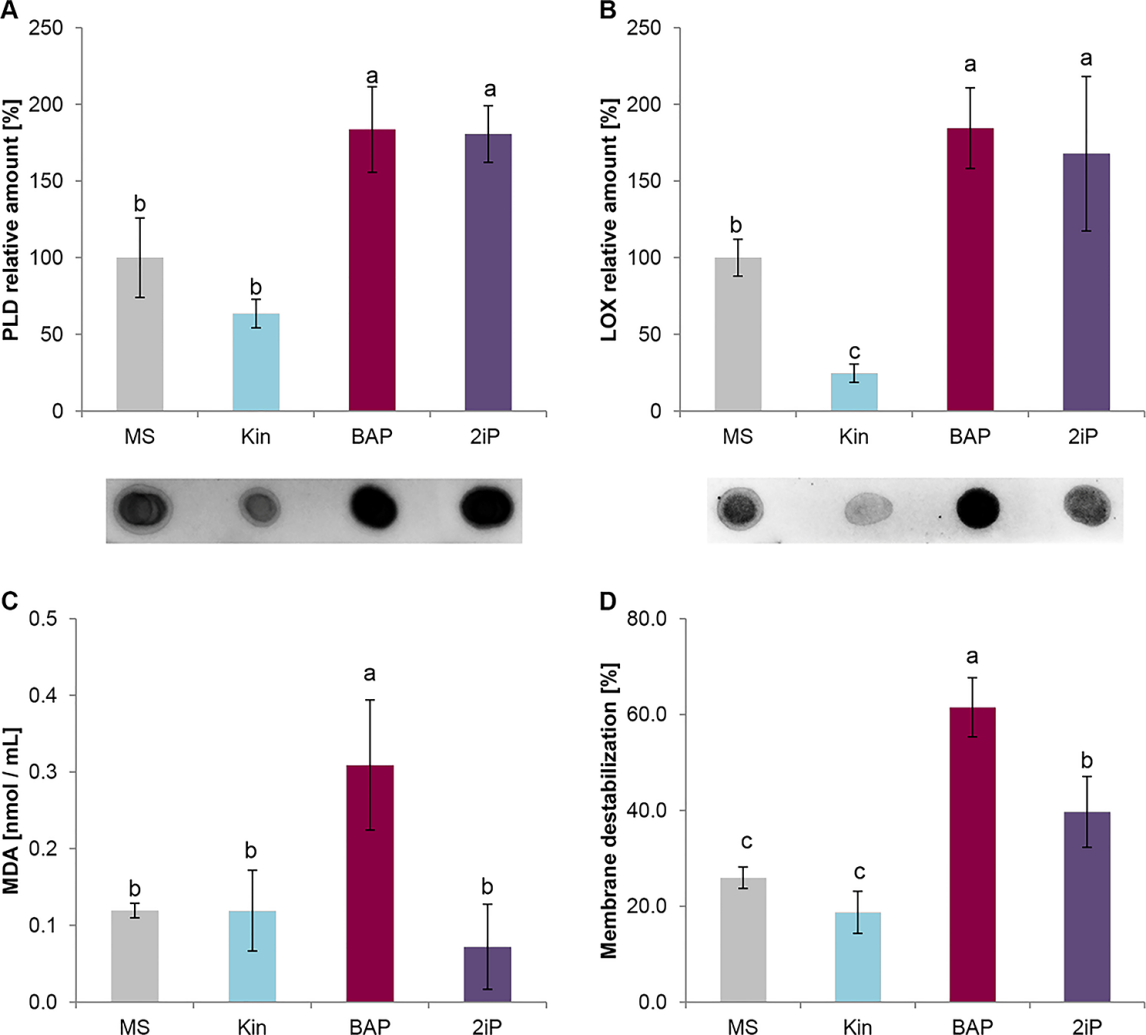
The relative amount of PHOSPHOLIPASE D (PLD; **A**) and LIPOXYGENASE (LOX; **B**), malondialdehyde content (MDA; **C**), and membrane destabilization (**D**) in kale leaves treated with different cytokinins under in vitro conditions. Kale microshoots were grown on MS medium without phytohormones and supplemented with different cytokinin at a concentration of 2.5 mg/dm3: kinetin (Kin), N6-(2-isopentenyl)adenine (2iP), 6-benzylaminopurine (BAP). Different letters imply significant differences (*p* ≤ 0.05, Tukey test)

BAP and 2iP, increased the amount of both PLD (Fig. 5A) and LOX (Fig. 5B), leading to a significant increase in membrane permeability. The strongest destructive activity was observed for shoots treated with BAP, where the MDA level and membrane permeability were almost three times higher than those of the control (0.31 ± 0.08 nmol/mL and 62 ± 6%, respectively; Fig. 5C and D). Among all the tested CKs, Kin was the only one that did not affect the cell membrane stability and even decreased the relative amount of LOX. Moreover, the MDA content and membrane permeability were comparable to the control.

### Steroids metabolism

In response to the CK presence, the activity of HMGR exhibited a significant two-fold increase, regardless of the CK type. The enzyme activity increased from 0.276 ± 0.022 µmol NADPH/min/mg protein in the control to 0.635 ± 0.009 µmol NADPH/min/mg protein for shoots treated with BAP (Table 1).

**Table 1:** Activity of 3-hydroxy-3-methylglutaryl-CoA reductase (HMGR) in kale leaves after treatment with various cytokinins under in vitro conditions. Kale microshoots were grown on MS medium without phytohormones and supplemented with different cytokinin at a concentration of 2.5 mg/dm^3^: kinetin (Kin), N6-(2-isopentenyl)adenine (2iP), 6-benzylaminopurine (BAP). Different letters imply significant differences (*p* ≤ 0.05, Tukey test)

Cytokinin	HMGR activity [µmol NADPH / min / mg protein]
MS	0.276 ± 0.022^b^
Kin	0.601 ± 0.085^a^
BAP	0.635 ± 0.009^a^
2iP	0.598 ± 0.094^a^

Confirmation of CK’s stimulatory effect on steroids was further verified through GC-MS/FID results. The total steroids content increased from 789.7 µg/g DW in the control to its highest value of 1871.5 µg/g DW after 2iP treatment (Table 2). Notably, 2iP treatment demonstrated the highest levels of cholesterol precursors, including cycloartenol and cholesterol itself, along with cycloartenol acetate as a potential reservoir of steroid precursors. The dominant sterols observed across all treatments were sitosterol and campesterol. The characteristic sterol of the Brassicaceae family, brassicasterol, was notably absent in kale leaves obtained on the medium supplemented with Kin. Furthermore, its presence in leaves from the other treatments revealed the lowest levels among all determined steroids. When contrasting this observation with the levels of campestanol, it becomes apparent that the saturation of campesterol is consistently favored in all treatments over its desaturation. A similar trend was observed for sitosterol, wherein its saturated form, sitostanol, exceeded the desaturated stigmasterol by several times. Additionally, a noteworthy abundance of sitostenone, a steroid ketone, was observed across all treatments, particularly high in shoots cultivated on medium with 2iP (110.9 ± 10.1 µg/g DW; Table 2).

**Table 2:** The content of steroids in kale leaves subjected to different cytokinins in vitro (kinetin, Kin; N6-(2-isopentenyl)adenine, 2iP; 6-benzylaminopurine, BAP). Different letters imply significant differences (*p* ≤ 0.05, Tukey test)

	Content [µg/g DW]			
compound	MS	Kin	BAP	2iP
cholesterol	12.6 ± 2.0^c^	29.2 ± 1.1^b^	17.5 ± 1.0^c^	73.8 ± 4.4^a^
cycloartenol	28.5 ± 1.6^c^	32.1 ± 2.8^bc^	38.1 ± 1.2^b^	61.1 ± 6.2^a^
cycloartenol acetate	44.0 ± 2.9^c^	63.3 ± 2.4^b^	48.7 ± 1.4^c^	70.4 ± 3.5^a^
campesterol	134.1 ± 7.0^b^	139.9 ± 11.1^b^	275.2 ± 18.2^a^	296.2 ± 20.9^a^
brassicasterol	5.3 ± 0.4^b^	n.d.	16.7 ± 0.9^a^	4.2 ± 0.3^b^
campestanol	28.1 ± 2.2^c^	51.4 ± 2.4^b^	29.5 ± 0.6^c^	58.7 ± 4.2^a^
sitosterol	398.5 ± 26.9^b^	426.2 ± 23.9^b^	932.1 ± 32.5^a^	1057.2 ± 93.1^a^
stigmasterol	19.6 ± 1.4^b^	20.7 ± 2.4^b^	29.9 ± 1.5^a^	32.9 ± 3.0^a^
sitostanol	54.2 ± 4.0^c^	113.4 ± 11.8^a^	72.3 ± 2.6^b^	106.1 ± 14.9^a^
sitostenone	64.8 ± 3.8^c^	67.7 ± 1.9^c^	90.1 ± 4.2^b^	110.9 ± 10.1^a^
*Sum of steroids*	*789.7 ± 38.2* ^*d*^	*943.7 ± 18.3* ^*c*^	*1549.9 ± 46.7* ^*b*^	*1871.5 ± 68.3* ^*a*^

n.d. – not detected

The ester and glycoside forms of sterols referred to cholesterol, brassicasterol, campesterol, stigmasterol, and sitosterol. Notably, the highest levels of both sterol derivatives were observed in shoots treated with 2iP, which correlated with elevated content of free sterols. Conversely, exogenous Kin significantly downregulated esters and glycosides, even when compared to the control plants, where the level of free sterols was higher than that of Kin-treated shoots (Tables 3 and 4).

**Table 3:** Levels of esters derivative of sterols in kale cultivated on medium enriched with various cytokinins (kinetin, Kin; N6-(2-isopentenyl)adenine, 2iP; 6-benzylaminopurine, BAP). Different letters imply significant differences (*p* ≤ 0.05, Tukey test)

	Content [µg/g DW]			
compound	MS	Kin	BAP	2iP
cholesterol	1.9 ± 0.1^a^	0.2 ± 0.0^d^	0.8 ± 0.1^c^	1.4 ± 0.2^b^
brassicasterol	1.7 ± 0.1^a^	n.d.	1.0 ± 0.1^b^	n.d.
campesterol	22.4 ± 2.4^b^	17.6 ± 0.9^b^	45.9 ± 5.3^a^	48.8 ± 3.5^a^
stigmasterol	8.1 ± 1.5^c^	6.5 ± 0.4^c^	12.7 ± 0.5^b^	16.0 ± 0.6^a^
sitosterol	54.4 ± 4.6^b^	40.5 ± 4.1^c^	99.9 ± 5.8^a^	107.1 ± 6.3^a^
*Sum of esters*	*88.5 ± 7.7* ^*b*^	*64.8 ± 3.6* ^*c*^	*160.3 ± 10.7* ^*a*^	*173.3 ± 4.7* ^*a*^

n.d. – not detected

**Table 4:** The content of sterol glycosides in kale treated with different cytokinins under in vitro conditions (kinetin, Kin; N6-(2-isopentenyl)adenine, 2iP; 6-benzylaminopurine, BAP). Different letters imply significant differences (*p* ≤ 0.05, Tukey test)

	Content [µg/g DW]			
glycoside derivative	MS	Kin	BAP	2iP
cholesterol	2.9 ± 0.2^a^	0.2 ± 0.0^c^	2.0 ± 0.1^b^	1.9 ± 0.1^b^
brassicasterol	2.4 ± 0.1^a^	n.d.	n.d.	2.6 ± 0.1^a^
campesterol	32.4 ± 2.1^c^	23.8 ± 1.2^d^	63.3 ± 1.9^a^	58.2 ± 2.4^b^
stigmasterol	11.9 ± 0.8^b^	7.5 ± 0.2^c^	20.0 ± 1.1^a^	13.6 ± 0.5^b^
sitosterol	82.5 ± 4.0^c^	58.7 ± 3.1^d^	147.1 ± 6.4^a^	117.0 ± 6.5^b^
*Sum of glycosides*	*132.1 ± 5.9* ^*c*^	*90.2 ± 2.6* ^*d*^	*193.3 ± 5.2* ^*b*^	*232.4 ± 6.0* ^*a*^

n.d. – not detected

### Brassinosteroid perception

**Fig. 6:**
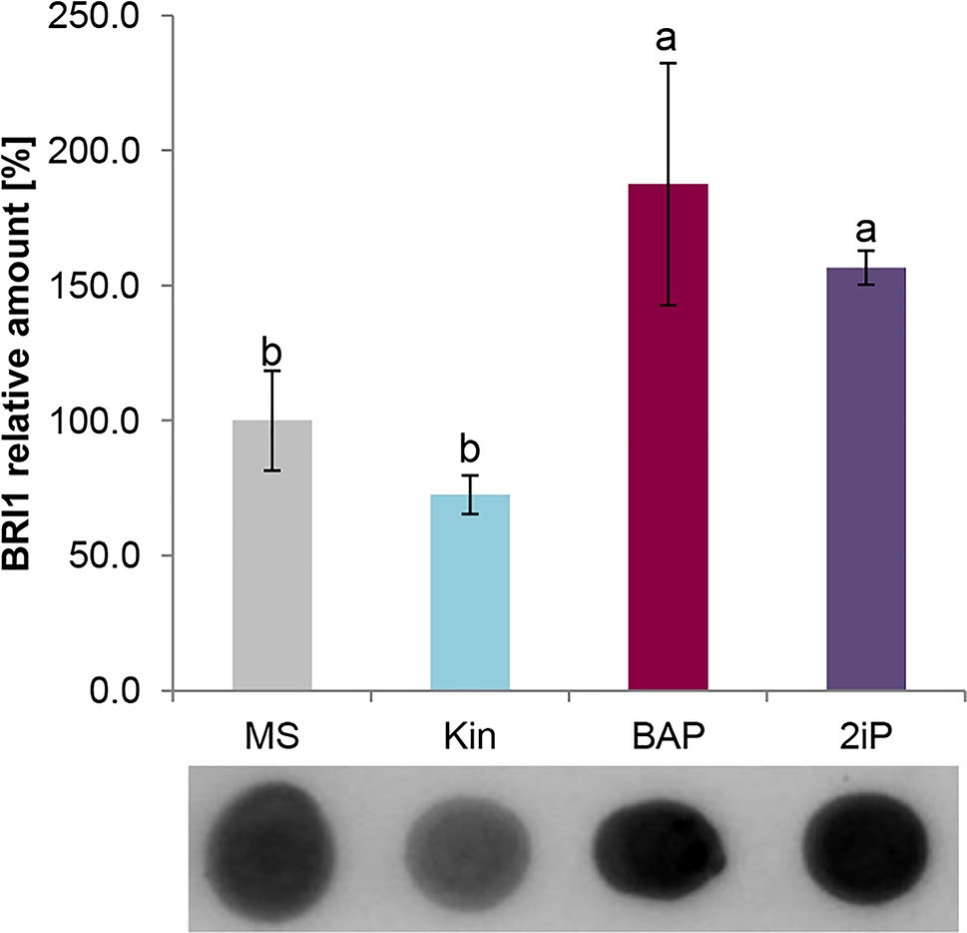
The relative amount of BRASSINOSTEROID INSENSITIVE1 (BRI1) in kale leaves after cytokinin treatment under in vitro conditions. Kale microshoots were grown on MS medium without phytohormones and supplemented with different cytokinin at a concentration of 2.5 mg/dm^3^: kinetin (Kin), N6-(2-isopentenyl)adenine (2iP), 6-benzylaminopurine (BAP). Different letters imply significant differences (*p* ≤ 0.05, Tukey test)

Given the differential amount of the initial compound for the synthesis of the BRs, in the next step we verify whether BR signaling is switched. Reaction with antibodies against BR receptor (anti-BRI1, BRASSINOSTEROID INSENSITIVE1) revealed that BAP and 2iP upregulated the relative content of this protein (Fig. 6). No effect has been observed under the treatment with Kin.

## Discussion

In recent studies, it was demonstrated that the impact of several CKs on kale differs under in vitro conditions leading to various responses, e.g. plantlets morphology and proliferation rate [20]. A direct consequence of the previous experiment was to gain knowledge about the mechanism of action of CKs selected as efficient for in vitro studies. That is why, here, we performed comprehensive analyses and provided considerable evidence for the differential action of Kin, BAP, and 2iP in the leaves of kale. A reduction in pigment content corresponded to enhanced shoot proliferation on BAP and 2iP medium. Interestingly, this coincided with the observation of the lowest values of the maximum quantum yield of PSII, suggesting a potential trade-off between shoot proliferation and photosynthetic efficiency in kale cells stimulated by CKs, particularly BAP. Nevertheless, the increased respiratory rate in CK-treated shoots indicates heightened metabolic activity, possibly to support the intensified rate of cell division [43]. Elevated respiratory rates often accompany increased energy consumption, potentially leading to higher levels of ROS. Nevertheless, only shoots treated with 2iP showed a significant accumulation of H_2_O_2_ compared to control plants. Conversely, both Kin and BAP treatments led to decreased H_2_O_2_ levels, with Kin even reducing O_2_^•-^content in plantlets. The presented findings prompted us to check the antioxidant status of kale. Shoots treated with Kin, consistent with ROS levels, exhibited the lowest activity of SOD and CAT, with comparable CAT activity also noted for shoots treated with BAP, which aligned with the decreased level of H_2_O_2_. In turn, GPOX, which utilizes H_2_O_2_ as a substrate, was significantly increased by all CKs, similar to findings obtained in micropropagated *Crocus sativus* [44].

The oxidative status is also influenced by the Foyer-Halliwell-Asada cycle, involving low-molecular-weight components such as Asc and GSH, as well as enzymes related to their metabolism. The antioxidative properties of kale extracts changed significantly in CK-specific manners. Accumulation of Asc in response to Kin could be an effect of MDHAR and DHAR action. In turn, enzymes involved in Asc oxidation (APX and AO) are not affected by Kin, which is in line with maintaining a high level of its reduced form. A possible explanation for the low GSH content might be the action of DHAR, which also uses GSH as a substrate and provides huge amounts of Asc. The response of kale to the BAP is more likely associated with the Asc-related part of the Foyer-Halliwell-Asada cycle since the activity of DHAR/GR and the GSH/GSSG content are not affected. Thus, the antioxidative properties of accumulated Asc are supported by higher activity of AO and, as a consequence, the formation of DHA. Under 2iP treatment, a strong burst of H_2_O_2_, significantly detoxified by GPOX, leads also to the production of antioxidants such as Asc and GSH. Analyzing this part, it should be noted that the redox balance of kale mediated by CK treatment is highly dependent on the Asc/DHA rather than GSH/GSSG. This is in agreement with previously published results, demonstrating that Asc and DHA play an important role in the regulation of mitotic activity in the meristems attributed to CKs. Particularly, Asc promotes cell-cycle progression in the root apical meristem of *Allium cepa* by stimulating the G1-S transition [45–47]. These ROS detoxifying potential of CK-related induction of endogenous small-molecular-weight antioxidants in plant explants could be useful for in vitro culture approaches oriented toward the production of these beneficial compounds.

The redox status can be also evaluated through the measurement of the nicotinamide adenine dinucleotides NAD and NADP, as well as their reduced forms, which reflect the overall metabolic activity of plant cells. CK notably elevated the content of NADH, NADPH, and NADP, while diminishing NAD concentration. Particularly, in shoots treated with Kin, NADH reached its peak level, which aligns with the heightened activity of MDHAR, utilizing NADH in reduction reactions to produce, e.g. Asc. Furthermore, the increased level of NADH in shoots treated with CKs correlates with a higher respiration rate, which could be in line with the reduction in NAD content.

Excessive production of ROS might lead to radical damage of cell membranes through the peroxidation of lipids, particularly polyunsaturated fatty acids (PUFAs). Deleterious effects on membrane structure have PLD and LOX - two key enzymes in the cascade of degradation of phospholipids and fatty acids [48]. The proliferative effect evoked by BAP and 2iP in kale, associated with the induction of PLD and LOX, indicates modification of lipid bilayer structure. Literature data suggests that PLD could provide sitosterol [49] and it is possible in kale given the great abundance of this compound in BAP and 2iP-treated plants. In turn, a high amount of LOX can be related to carotenoid degradation, as documented also in tomato [50]. Grossman and Leshem [51] found that exogenous Kin reduced the activity of LOX in pea, which could explain the lower content of this enzyme in kale under the action of this CK. LOX oxidizes PUFAs to peroxide products and MDA [52], suggesting that the MDA burst in BAP-treated kale is a consequence of LOX-mediated generation of peroxidative damage in the lipid bilayer, confirmed by the increased percentage of membrane destabilization. A higher content of MDA under in vitro conditions was also reported for *Allium schoenoprasum* [53].

Membrane properties are maintained by specific compounds known as sterols, which regulate the dynamic of the microfluid state. Upregulation of HMGR activity after treatment with all CKs supports the involvement of this enzyme in growth promotion, as previously shown [54], and suggests intensification of de novo sterols synthesis. This hypothesis was verified using GC-MS, based on which we observed a strong increase in sterol content in kale’s response to CKs. In general, accumulated phytosterols reduce the intestinal absorption of cholesterol and thus help maintain cardiovascular health and have become beneficial to the biotechnological industry, especially with regard to the production of various food additives [13]. Based on the above reasons, we conclude that CKs-dependent in vitro cultivation of kale is a promising method for the production of beneficial steroid compounds.

BAP and 2iP treatment leads to the accumulation of cycloartenol (a key precursor in the synthesis of other phytosterols) as well as stigmasterol, which is required for cell proliferation and differentiation [55]. Notably, both CKs demonstrated the most potent stimulatory effect on steroid formation, especially campesterol, stigmasterol, and sitosterol, involved in the regulation of membrane fluidity and permeability through the inhibition of the mobility of fatty acyl chains [56]. Sitosterol also contributes to cellulose biosynthesis [57], which might explain its highest abundance, greatly enhanced by all CKs treatments in kale extracts. Sitosterol could be necessary for the formation of cell walls of newly divided cells under the action of phytohormones. In medicine, sitosterol indicates a valuable effect on slowing cell development and even triggers cell death in cancers such as prostate, breast, liver, colon, and murine fibrosarcoma cells. Thus, the proposed methodology of in vitro propagation can increase the productivity of kale and serve as a template for the chemical components for the synthesis of novel anti-tumor drugs [58].

Our results suggest that CK-treated kale showed a higher content of saturated forms of campesterol and sitosterol (campestanol and sitostanol) compared to unsaturated brassicasterol and stigmasterol. This increase in saturated forms may be due to the strengthening of NADPH levels, which are possibly involved in the decline in these steroidal substrates. Previous research already indicated a tendency of plant cultures to reduce the double bond of steroidal substrates in NADPH-dependent manner [59]. Accumulated sitosterol, as well as stigmasterol and cholesterol under CKs treatment in kale, might be important component of specific membrane regions - lipid microdomains (rafts) critical for fundamental biological processes such as signaling, cellular sorting, or cytoskeleton reorganization [60] important for cells proliferation. Furthermore, this study highlights that both BAP and 2iP foster the accumulation of sitostenone (a derivative of sitosterol and stigmasterol) recognized for its antioxidant properties, capacity to enhance glucose uptake and ameliorate insulin resistance in hyperglycemia-induced hepatic cells in vitro [61]. It underscores the potential therapeutic benefits of CKs.

An interesting finding of the presented study is the differential action of various CKs on the level of brassicasterol – the precursor of BRs synthesis. It suggests interactions among CKs and BRs under in vitro similarly as shown previously in rice [62]. To verify such hypothesis in kale we performed biochemical analyses using anti-BRI1 antibody recognizing receptor of BRs. Accumulation of brassicasterol is accompanied by upregulation of receptor protein which supports induction of BRs signal transduction pathway. No direct correlation between sterol and receptor content was noted for 2iP treatment. A possible explanation for the accumulation of BRI1 and at the same time no change in brassicasterol could be related to the perception of BRs appearing due to hydrolysis of conjugated BRs forms rather than *de novo* biosynthesis of these hormones. Critical for the optimal function of BRs is the maintaining of homeostasis by catabolism reactions, e.g. hydroxylation or glucosylation, which decreases the level of active hormones and attenuates signaling output [63]. To the best of our knowledge, this is the first report about the analysis of the BR1 receptor in plants cultivated under in vitro conditions. Correlation among BRI1 level and valuable effect on the in vitro propagation rate indicates a cross-talk between CKs and BRs and could be useful for further analysis oriented toward improvement of methodology of Brassicaceae cultivation under specific conditions, independently from climatic factors.

The profile of sterols conjugates in kale revealed that among all identified forms five of them (cholesterol, brassicasterol, campesterol, stigmasterol, and sitosterol) seem to undergo the reaction of esterification and glycosylation. However, it implies that kale’s ability to proliferate under BAP and 2iP treatment requires only the conversion of campesterol, stigmasterol, and sitosterol into esterified and glycosylated forms. These observations might be in line with the findings of Ferrer et al. [64], who suggest that sterol esters serve as a storage reservoir for sterols that can be used during growth and development. In kale, as membrane destabilization escalates, there is a concurrent rise in the level of sterol glycosides, which can most likely play a vital function in protecting cell membrane integrity, as evidenced in *Solanum* species [64]. Interestingly, based on the results presented here, sterol turnover via esterification/glycosylation reaction is not a strategy of kale induced by Kin, given the reduced content of sterol esters and glycosides.

Regarding differences among the applied CKs, although we used CKs characterized by various chemical structures: both aromatic (Kin, BAP) and isoprenoid (2iP), based on the majority of results it is hard to distinguish a direct relationship between the type of compound and physiological/biochemical response. However, isoprenoid 2iP is most effective in the promotion of sterols accumulation and antioxidant activity (APX, GSH) in kale without strong induction of proliferation as observed in the case of aromatic BAP. This fact can be used for the selection of CKs for research-oriented on different aims: multiplication of plant material or synthesis of beneficial compounds.

## Conclusion

In this study, we provide a physiological background of the CKs’ action in kale cultivated under in vitro conditions. Special attention was paid to antioxidants, lipids, and sterols and these results were deeply discussed in relation to membrane functioning. CKs effectively stimulate sterol accumulation which affects membrane properties and enhances Asc metabolism related to oxidative status, supporting a complex biochemical response. Based on the presented results we conclude that the proliferative effect evoked by BAP and 2iP improves the industrial significance of kale. Our future studies are oriented toward performance of molecular analyses, such as NGS experiments which will be helpful for describing candidate genes and metabolic pathways that are induced by exogenous CKs in kale under in vitro cultivation. Modulation of expression of these specific genes can help to accelerate proliferation rate. The proposed methodology of cultivation gives also the possibility to control biosynthetic routes for obtaining desired beneficial compounds such as antioxidants or nutraceuticals. What is more, the obtained results might be useful for the optimization of propagation techniques of other Brassicaceae species and this can bring significant environmental and economic advantages.

## Data Availability

The raw data of the presented results of this study are available on request to the corresponding author.
